# Association of the miR-143 Gene rs353292 Polymorphism with Recurrent Pregnancy Loss in Caucasian Women: A Novel Finding in a Multifactorial Devastating Problem

**DOI:** 10.3390/ijms252211952

**Published:** 2024-11-07

**Authors:** Sofoklis Stavros, Anastasios Potiris, Panagiotis Christopoulos, Natalia Zacharopoulou, Vasiliki Kyrli, Despoina Mavrogianni, Athanasios Zikopoulos, Eirini Drakaki, Theodoros Karampitsakos, Spyridon Topis, Nikolaos Machairiotis, Angeliki Gerede, Chara Skentou, Peter Drakakis, Ekaterini Domali

**Affiliations:** 1 Third Department of Obstetrics and Gynecology, University General Hospital “ATTIKON”, Medical School, National and Kapodistrian University of Athens, 12462 Athens, Greece; sfstavrou@med.uoa.gr (S.S.); thanzik92@gmail.com (A.Z.); theokarampitsakos@hotmail.com (T.K.); spyrostopis@med.uoa.gr (S.T.); nikolaosmachairiotis@gmail.com (N.M.); pdrakakis@med.uoa.gr (P.D.); 2 Second Department of Obstetrics and Gynecology, Aretaieion University Hospital, Medical School, National and Kapodistrian University of Athens, 11528 Athens, Greece; panchrist@med.uoa.gr; 3 First Department of Obstetrics and Gynecology, Alexandra Hospital, Medical School, National and Kapodistrian University of Athens, 11528 Athens, Greece; zacharopoulou.nat@gmail.com (N.Z.); kyrlivaso@gmail.com (V.K.); dmavrogianni@med.uoa.gr (D.M.); eirinidrak@med.uoa.gr (E.D.); kdomali@yahoo.fr (E.D.); 4 Department of Obstetrics and Gynecology, Democritus University of Thrace, 69100 Campus, Greece; agerede@otenet.gr; 5 Department of Obstetrics and Gynecology, Medical School, University of Ioannina, 45110 Ioannina, Greece; haraskentou@uoi.gr

**Keywords:** miR-143 gene, rs353292 polymorphism, recurrent pregnancy loss (RPL), miscarriage, polymorphism

## Abstract

The purpose of this prospective case–control study is to investigate the correlation of the miR-143 gene rs353292 polymorphism in Caucasian women with recurrent pregnancy loss (RPL) compared to a matched control group with at least one live birth and without pregnancy losses. In total, 110 women with recurrent pregnancy losses and 95 control women were recruited. Peripheral blood was collected from all women, and the isolation of DNA was performed with Monarch Genomic DNA Purification. Polymerase chain reaction was applied to amplify the DNA sequence of the miR-143 gene promoter, carrying the polymorphism rs353292. The incidence of genotype CC in the RPL group was statistically significantly higher than in control group (*p* < 0.0001). Allele C (CT + CC) in the control group was found in 47.36%, and in the RPL group was found in 68.17% (*p* = 0.006). SNP rs353292 T>C was associated with increased risk of recurrent pregnancy loss. The calculated odds ratio for CT + CC vs. TT and for CC vs. TT were significant higher (*p* = 0.0028 and *p* < 0.0001, respectively). The study results suggest that the rs353292 polymorphism is associated with a statistically significant increase in RPL prevalence. The present study provides additional evidence in favor of a shared pathophysiological mechanism that contributes to both RPLs, potentially through inflammatory processes and epithelial–mesenchymal transition dysregulation.

## 1. Introduction

Pregnancy loss is one of the most common complications in pregnancies and refers to the spontaneous demise of a pregnancy before the embryo reaches the viability threshold [1]. The loss of two or more consecutive pregnancies defines the term recurrent pregnancy loss (RPL). At least 25–50% of women will experience one or more miscarriages, and the main etiologic factors include endocrine dysfunctions, anatomic and environmental factors, antiphospholipid syndrome, thrombophilia, autoimmune diseases, infections, and genetic factors [2,3].

The average prevalence of anatomic factors in the general population is about 4.3%, in infertile patients about 3.5%, and in patients with recurrent pregnancy losses about 13% [4,5]. About 17–20% of recurrent miscarriages are related to endocrine abnormalities, such as luteal phase deficiency (LPD), polycystic ovary syndrome (PCOS), diabetes mellitus, thyroid disease, and hyperprolactinemia. Advanced maternal age is a significant factor, as it increases the likelihood of chromosomal abnormalities and reduces oocyte quality. Lifestyle factors, including smoking, excessive alcohol consumption, and high stress levels, can also contribute to recurrent pregnancy loss [6]. Genetic factors are associated with 30–50 percent of RPLs [7]. Several studies have highlighted the relationship between cardiovascular diseases and recurrent miscarriages, suggesting that common risk factors may be involved. One of the possible factors may be endothelial dysfunction, which can damage the development of the uteroplacental vasculature, which is important for fetal development and is also responsible for hypertension and metabolic syndrome [8]. Maternal endothelial dysfunction may impair the invasion of extravillous cytotrophoblasts into the spiral arteries, which are essential to the establishment of the high-flow low-resistance uteroplacental system that provides the blood supply for fetal development [9]. Coagulation disorders have been reported as risk factors for recurrent miscarriages and cardiovascular diseases [10]. Therefore, many studies focus on thrombophilic and other genes, which may also be related to recurrent miscarriages [11].

MicroRNAs play a key role in regulating gene expression, impacting processes such as cell growth, differentiation, and apoptosis. The rs353292 polymorphism of the miR-143 gene is a single nucleotide polymorphism (SNP) in the promoter region, and it involves a substitution of cytosine (C) with thymine (T). This genetic variation can alter miRNA expression, thereby impacting downstream genes involved in various processes potentially critical to pregnancy maintenance. SNPs in miRNA genes have been found to be associated with recurrent miscarriages [12]. Recent evidence shows the presence of a highly conserved region of the promoter of the miR-143/145 complex, which contains binding sites for transcription factors that regulate vascular smooth muscle cell (VSMC) functional contractility [13]. Studies have shown that microRNA-143 is involved in the development and progression of various cardiovascular diseases such as atherosclerosis, arterial hypertension, and coronary heart disease [13,14,15]. The rs353292 polymorphism was positively associated with chronic kidney disease (CKD), and a significant association was observed between CC + TC and TT genotypes and CKD stages [16]. Moreover, the rs353292 polymorphism was associated with an increased risk of developing colorectal cancer in heterozygous comparison and CT/TT carriers exhibited a lower expression in miR-143 compared to the CC carriers [17]. Ultimately, the rs353292 polymorphism of the miR-143 gene is gaining attention in the field of reproductive health and in recurrent pregnancy loss [18].

The aim of this study is the investigation of a possible correlation between the rs353292 polymorphism of the miR-143 gene in Caucasian women and recurrent pregnancy losses, compared to a matched control group with at least one live birth and without any previous pregnancy losses.

## 2. Results

### 2.1. Baseline Characteristics

Table 1 summarizes the baseline characteristics for the recurrent pregnancy loss and control groups. In total, 110 women with RPL and 95 controls were included in our study. Mean age was 33.95 for the RPL group and 34.01 for the control group, while the mean values of body mass index (BMI) for both groups were 23.08 and 23.27, respectively. Both age and BMI did not differ between the two groups.

### 2.2. rs353292 Gene Polymorphism in Recurrent Pregnancy Loss and Control Groups

The genotype frequencies in females with RPL and in the control group are shown in Table 2. For rs353292, the most frequent genotype in the control group was TT (52.63%) and in the RPL group was CC (34.54%). Genotype CC in the control group appears in 3.15%, and in the RPL group appears in 34.54% (*p* < 0.0001). However, allele C (CT + CC) in the control group was 47.36% and in the RPL group was 68.17% (*p* = 0.006). SNP rs353292 T>C was associated with increased risk of RPL. The calculated odds ratio for CT + CC vs. TT 2.38 (95% CI 1.35–4.20, *p* = 0.0028), and for CC vs. TT 17.62 (95% CI 5.03–61.70, *p* < 0.0001).

## 3. Discussion

The association of polymorphisms with recurrent pregnancy loss has been studied extensively. Polymorphisms located in miRNA-143 may cause changes in expression patterns, and have also been associated with cardiovascular diseases [17,19]. From the data analysis for SNP rs353292, it is revealed that in women with RPL, the C allele is detected more often (68.17%) compared to controls (47.36%). The odds ratio was calculated as 2.38 and 95% CI (1.34–4.20). Therefore, women carrying the C allele are 2.38 times more likely to have recurrent miscarriages than women carrying the T allele (*p* = 0.028). In alignment with our results is a recent study that considers the miR143 polymorphism (rs353292, T>C) as a risk factor for recurrent miscarriages [18]. However, it should be mentioned that the study from Ezat et al. included an Iraqi population and ours included a Caucasian white Hellenic population.

In women with unexplained recurrent miscarriages, the secretion of IFN-γ and TNF-α from NK cells has been found to be significantly higher than in normal pregnancies. This suggests the involvement of the MAPK signaling pathway [20]. Moreover, the levels of IL-1β in the placenta of women with spontaneous abortions have been observed to be elevated [21]. Additionally, a study found that women with a history of miscarriage exhibited elevated levels of IL-6, IL-17, and TGF-β compared to the control group. This suggests that women with recurrent miscarriages show increased immune activity, locally and systemically, irrespective of pregnancy status [22]. It is indicated that miR-143-3p significantly inhibits the production of IL-1β, IL-6, and IL-8 [23]. Additionally, in miscarriages, an overexpression of miR-143 has been found [24]. It could be hypothesized that the presence of rs353292 may lead to the overexpression of miR-143 and, consequently, to inflammatory conditions related to recurrent pregnancy loss.

The miR-143 gene has been implicated in recurrent pregnancy loss due to its regulatory role in placental development and function via the regulation of epithelial–mesenchymal transition (EMT), during which the epithelial cells lose polarity and cell–cell adhesion properties and acquire migratory and infiltrative properties to become mesenchymal cells [25]. The EMT contributes to the invasion of the blastocyst into the uterus and the subsequent proper anchoring of the placenta [26]. Abnormal processes in the epithelial–mesenchymal transition can cause implantation failure and placental pathologies, including pre-eclampsia and placenta accreta/increta/percreta [27,28]. Research suggests that miR-143 might influence recurrent pregnancy loss by targeting genes involved in the vascularization of the endometrium, which is crucial for successful implantation. Furthermore, the dysregulation of miR-143 has been linked to increased apoptosis in trophoblast cells, impairing the placenta’s ability to support fetal development and leading to adverse pregnancy outcomes [29,30,31]. miR-143 has been identified as a key player in the mechanism of epithelial–mesenchymal metaplasia. In breast cancer, it has been demonstrated that the elevated expression of miR-143 results in the suppression of migration and proliferation of breast cancer cells through ERK5 [32]. In RPL, it has been demonstrated that epithelial–mesenchymal metaptosis is suppressed. In particular, trophoblastic cells lose this ability, which consequently prevents implantation from occurring [33]. Additionally, altered expression of miR-143 has been associated with the dysregulation of key signaling pathways that affect embryo viability and maternal–fetal communication, potentially contributing to the risk of repeated pregnancy loss. The inhibited activities of trophoblast cells in relation to EMT are closely related to the pathogenesis of RPL.

A recent investigation into the mechanisms of action of miR-143 in unexplained recurrent miscarriages demonstrated that miR-143-3p inhibited trophoblast cell viability, induced apoptosis, and reduced the invasion and migratory capacity of these cells. This was achieved by suppressing the epithelial–mesenchymal transition process. Increased miR-143-3p expression resulted in decreased levels of N-cadherin and vimentin while increasing E-cadherin levels. Conversely, decreased miR-143-3p expression exhibited the opposite effects [34]. Consequently, it could be hypothesized that the presence of the polymorphism is associated with a high expression of miR-143, leading to decreased EMT.

## 4. Materials and Methods

### 4.1. Study Design

This prospective case–control study included women who attended the Recurrent Miscarriage Department and the Assisted Reproduction Unit at the Alexandra Hospital, First Department of Obstetrics and Gynecology, Medical School of the National and Kapodistrian University of Athens. The sample was collected in a two-year period up to December 2023.

Our sample consisted of 110 women in the recurrent pregnancy loss group and 95 women in the control group. To be eligible for the recurrent pregnancy loss group, each participant should have at least two or more consecutive pregnancy losses. On the contrary, the inclusion criteria for the control group included at least one live birth without any prior pregnancy loss. Patient characteristics, such as age and body mass index (BMI), were registered for the RPL group. The age and the BMI of the control group were matched to those in the RPL group. Exclusion criteria included female participants with any endometrial or endocrinological pathology and/or any medical history of endometriosis, hydrosalpinx, autoimmune disorders, or chromosomal abnormalities. Data were not available for all partners and, consequently, were not included.

The estimation of the study sample size was based on the pilot results of the study, with the assumed incidence of allele C (CT + CC) in the control group being 47% and in the study group being 68%. The power analysis results were estimated for an alpha value of 0.05 and a power of 85% to avoid type I and type II errors. With those values, the sample was calculated at 198 participants, divided in two groups, namely the study group (*n* = 108) and the control group (*n* = 90), with an enrollment ratio of 1.2. The post hoc power analysis for our specific sample of 205 patients (110 in the recurrent pregnancy loss group and 95 in the control group) had a statistical power of over 99%.

### 4.2. Ethical Approval

This study was conducted in accordance with the Declaration of Helsinki and was approved by the Institutional Review Board of the Medical School of the National and Kapodistrian University of Athens with protocol identifier 91970. All of the patients included gave informed consent for their participation in this study. This study was conducted in the Assisted Reproduction Unit and Recurrent Miscarriage Department of the First Department of Obstetrics and Gynecology in Alexandra Hospital.

### 4.3. DNA Isolation and Genotyping of rs353292

Peripheral blood was collected from all women, and the isolation of DNA was performed with a Monarch Genomic DNA Purification Kit from New England Biolabs, Ipswich, MA, USA. Polymerase chain reaction (PCR) was applied to amplify the DNA sequence of the miR-143 gene promoter, carrying the polymorphism rs353292. Master Mix from New England Biolabs in 1× final concentration was used with 10 pmol of each primer and 2 µL of DNA to a final volume of 25 μL. The PCR conditions were 10 min at 94 °C and then for 35 cycles at 94 °C for 1 min, 55 °C for 1 min, and 72 °C for 1 min. The samples were incubated for 10 min at 72 °C. The sequences of the primers used were:

Forward 5′ CTTTCTTCTGCCACTCCTCCT 3′
Reverse 5′ GAAGGGCTTCAGAAT TCCG 3′

**Figure 1:**
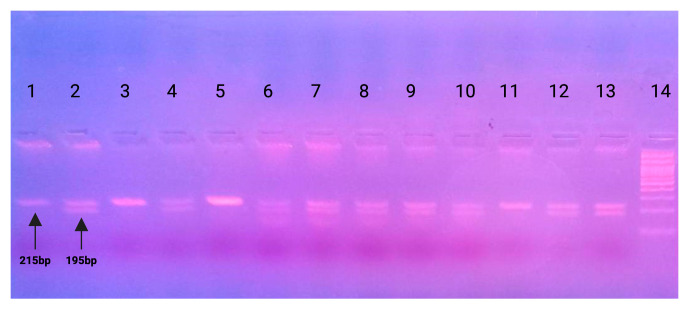
Detection of the rs353292 polymorphism using gel electrophoresis. Lanes 1, 3, 5, 11: Homozygosity for TT 215bp, Lanes 2, 4, 6, 7, 8, 9, 10, 12, 13: Heterozygosity 215 bp + 195 bp + 20 bp and Lane 14: 100 bp ladder.

All PCR products were visualized in UV after agarose electrophoresis. The restriction endonuclease BslI was used to detect the rs353292 polymorphism. In women carrying the polymorphism, Bsl1 will recognize the sequence in the PCR product and two fragments of 20 bp and 195 bp will be created. In women who do not carry the polymorphism, the enzyme will not recognize the restriction site and a 215 bp fragment will result. Figure 1 illustrates the detection of the rs353292 polymorphism using gel electrophoresis.

## 5. Conclusions

To our knowledge, our study is among the first that report the incidence and the effects of the rs353292 polymorphism of the miR-143 gene in recurrent pregnancy loss. This article is a primary attempt to detect if the rs353292 polymorphism can be used as a potential biomarker for recurrent pregnancy loss by applying molecular biology techniques. Our results suggest that the rs353292 polymorphism is associated with a statistically significant increase in RPL prevalence. The present study provides additional evidence in favor of a shared pathophysiological mechanism that contributes to both RPLs, potentially through inflammatory processes and epithelial–mesenchymal transition dysregulation. The strengths of the present study are the size of sample and the comparison with a matched control group. The limitation of the present study is the selection of only Caucasian white women. It is important to mention that our results reflect the incidence of the rs353292 polymorphism in the Hellenic population, and further studies are needed to extrapolate our results to the general population.

## Figures and Tables

**Table 1:** Baseline characteristics in RPL patients and control group.

Variable	Recurrent Pregnancy Loss Group *n* = 110	Control Group *n* = 95	*p*-Value
**Age (years)**			
Mean (SD)	33.95 (6.002)	34.01 (5.852)	0.946
Median (Q1, Q3)	33 (30, 38)	33 (30, 38)	
**BMI (kg/m^2^)**			
Mean (SD)	23.08 (3.254)	23.27 (3.306)	0.674
Median (Q1, Q3)	22.59 (20.34, 24.98)	22.59 (20.44, 25.00)	

**Table 2:** Genotype frequencies of women in the recurrent pregnancy loss group and control group.

SNP miR-143	Genotype	Control *n* (%)	RPL *n* (%)	*p*-Value	OR (95% CI)	OR *p*-Value
rs353292	TT	50 (52.63)	35 (31.81)	0.11	1.00	
	TC	42 (44.21)	38 (33.63)	0.65	1.29 (0.70–2.40)	0.41
	CC	3 (3.15)	37(34.54)	<0.0001	**17.62** (5.03–61.70)	<0.0001
	CC + TC	45 (47.36)	75 (68.17)	0.006	**2.38** (1.35–4.20)	0.0028
HWE *p*-value		0.1	0.05			

RPL: recurrent pregnancy loss, OR: odds ratio, CI: confidence interval, HWE: Hardy–Weinberg Equilibrium. Bold numbers indicate significant *p*-values.

## Data Availability

The raw data supporting the conclusions of this article will be made available by the corresponding author on request.
